# Unexplored avenues: a narrative review of cognition and mood in postmenopausal African women with female genital circumcision/mutilation/cutting

**DOI:** 10.3389/fgwh.2024.1409397

**Published:** 2025-01-09

**Authors:** Rohina Kumar, Noelia Calvo, Gillian Einstein

**Affiliations:** ^1^ Department of Psychology, University of Toronto, Toronto, ON, Canada; ^2^ Institute for Life Course & Aging, University of Toronto, Toronto, ON, Canada; ^3^ Tema Genus, Linköping University, Linköping, Sweden; ^4^ Rotman Research Institute, Baycrest Centre and University of Toronto, Toronto, ON, Canada; ^5^ Dalla Lana School of Public Health, University of Toronto, Toronto, ON, Canada; ^6^ Women’s College Research Institute, Toronto, ON, Canada

**Keywords:** female genital cutting, cognition, mood, postmenopausal women, African women, ageing, memory

## Abstract

Recent ageing research has projected the lifespan and proportion of postmenopausal women living in low- and middle-income countries to substantially increase over the years, especially on the African continent. An important subgroup within the African postmenopausal population is those with female genital circumcision/mutilation/cutting (FGC). Practised across 31 African nations, FGC holds cultural significance as it is deemed essential to marriage and successful womanhood. Perhaps because of this, most FGC studies have primarily focused on women's reproductive functioning and their mood experiences. These studies also usually exclude postmenopausal women from their cohorts. Consequently, cognition and age-related cognitive decline and preservation remain understudied. Therefore, we investigated what is known about mood and cognition in local and immigrant postmenopausal African women with FGC. To do this, we carried out a narrative review searching PubMed, PsycInfo, and Google Scholar databases. Boolean combinations of keywords related to FGC, cognition, ageing, and mood were used, with a focus on cognition and ageing-related terms. Only studies published in English, those that recruited African women with FGC aged 50 years and older, and those that investigated cognitive and/or mood-related experiences were included. Ten studies were found; these included quantitative, qualitative, and case reports. The age range of cohorts across included studies was 13–90 years; women who were likely postmenopausal formed a minority within the cohorts (4.5%–25%). There were no studies assessing memory or cognition beyond those looking at FGC-related memories, which were vivid, especially if women had type III FGC (Pharaonic) or were older at the time of FGC. Although most of these women reported experiencing negative emotions concerning FGC, quantitative reports showed that only a minority of women experienced post-traumatic stress disorder, anxiety, or depression. Thus, there remains an urgent need to bring this understudied group into ageing and dementia research. Future research should adopt mixed-methods with culturally sensitive methodologies to investigate the lived experience of ageing as well as cognitive changes. A holistic understanding of ageing women from the Horn of Africa's experiences and needs will support an improvement in the quality of care delivered to this cohort in both local and immigrant contexts.

## 1 Introduction

Greater than any other region worldwide, recent reports and population ageing studies have shown a dramatic increase in the average life expectancy in Africa (1); epidemiological research has shown that approximately 212 million adults 60 years and older will be living on the continent by 2050 (2). Concomitantly, mixed-sex and community-based studies have already shown evidence of cognitive decline in Africans over 2 years of follow-up (3) and increasing rates of dementia (4). Postmenopausal women form an important part of the African population, given that, by the late 2020s, the proportion of postmenopausal women living in developing countries is estimated to become 76% (5), with approximately 5 million of them living in sub-Saharan Africa (6). Some studies on sub-Saharan African women have shown the transition into the postmenopausal phase to be associated with a decline in cognitive function (6, 7). Outside of African contexts, studies have also shown that Alzheimer disease (AD) and related dementias (ADRDs) may disproportionately affect African American populations with increasing age (8). Moreover, African persons' experiences with racial discrimination in new Western contexts may predict a myriad of challenges with age, such as a higher risk of ADRDs (9) and accelerated rates of cognitive decline relative to their White counterparts (10).

In certain African countries, studies have indicated the presence of an increasing mental health burden among the ageing population (11). In general, ageing can be accompanied by an increased risk of depression; additionally, depression increases health problems and rates of mortality among older adults (12, 13). Multiple systematic reviews and meta-analyses have established a bi-directional relationship between depression and frailty (14), where one is associated with an increased incidence in the other, or one may be a risk factor for the development of the other (15–17). Similarly, research has also shown various mood disorders (e.g., major depressive disorder, bipolar disorder) to be major risk factors for AD, with this risk doubled among older adults (18, 19). This is likely because both mood disorders and AD have shared biological mechanisms (18). Mixed sex studies have shown faster rates of ageing for African Americans at risk of depression due to racial discrimination relative to those not at risk of depression (20), highlighting an undeniable influence of mood on their ageing experiences. Studies on postmenopausal local African women have also shown a high prevalence of depressive mood, irritability, and anxiety (21). An important subgroup of postmenopausal women in Africa includes those that have experienced female genital cutting/circumcision/mutilation (FGC); however, the links between menopause, FGC, cognition, and mood remain understudied.

FGC is a practice involving partial or total cutting/removal of the external female genitalia (22). There are variations in the type of FGC performed both within and across countries. Based on the extent of the cut/removal, a widely accepted classification system has been devised, consisting of four major FGC types (23). Type I FGC involves the partial or complete removal of the clitoral glans and/or the clitoral head, type II FGC involves partial or complete removal of the clitoral glands and labia minora (with or without removal of the labia majora), type III FGC involves the narrowing of the vaginal opening by cutting the labia minora/majora and suturing them (Pharaonic), and type IV FGC includes all other procedures done to the female genitalia for non-medical reasons (e.g., pricking, cauterization, piercing, etc.). Across 31 countries, at least 200 million girls and women have FGC, with the practice being most widespread in Northern Africa (24). Indeed, some of the highest FGC prevalence rates worldwide have been found in African countries such as Somalia, Djibouti, and Mali (25, 26). FGC is practised in diverse ways both between and within different ethnoracial groups (27).

Despite the ongoing debate about the ethical implications of FGC (28, 29); at present, the World Health Organization has recognised the practice as a form of violation of human rights, discrimination based on gender, and violence against girls (30). However, it is important to embed the practice in an African cultural context, given that it holds paramount significance in the lives of many African women, both locally and for those who immigrate. FGC marks their initiation into womanhood, instantiates femininity, and inscribes several values of comportment and aesthetics. The ritual allows them to physically resemble other members of their community and thus, be considered more marriageable, beautiful, hygienic, and pure (31–34). Moreover, many members of their community emphasise upholding the long-standing tradition to maintain their cultural heritage (35). If lacking FGC, these women may face sexual dishonour and risk being ostracised by members of their community (27, 36). Specific traditional components of FGC (e.g., age, environmental, and social factors) tend to vary based on different regions in Africa (37, 38); however, where practised, culture gives meaning and importance to the practice. Given that women live with FGC for the rest of their lives, it is critical for their health to understand the cognitive and mood experiences of ageing with FGC.

To date, most literature investigating the lived experiences of local and immigrant women with FGC has primarily focused on their reproductive health outcomes (39). Systematic reviews and meta-analyses have found factors such as prolonged labour, caesarean sections, difficult delivery, menstruation challenges, and pain during intercourse to be associated with FGC (40–42). Articles focusing on long-term complications post-FGC have also mainly investigated gynaecological, perinatal, and postnatal complications (43, 44). To our knowledge, only two papers focus on the possibility of long-term chronic pain and one on heart disease, independent of reproduction - all from our research group (45–47). Due to this narrow focus on reproduction, most studies tend to recruit women with FGC in their reproductive or childbearing years, often ranging from 15 to 49 years (e.g., 48, 49). Thus, there still remains a scarcity of research conducted on any other health aspect beyond women with FGC's reproductive health (47).

Previous reviews focusing on women with FGC's mental health outcomes reported that they often have high incidences of affective disorders or post-traumatic stress disorder [PTSD; (50)]. Similarly, a systematic review found that women with FGC tend to report a higher burden of adverse mental health outcomes relative to women without FGC (51). In relation to cognition, there was only one pilot study that investigated the mental health status and memory of local Senegalese women of reproductive age with and without FGC. They found a diagnosis of PTSD to be associated with the presence of significant memory problems among young women with FGC relative to those without FGC (52). However, these findings are controversial since some studies have not found significant differences in psychological consequences between women with FGC and those without (53). None of these reviews or the one pilot study looked at cognition in the absence of some pathology purportedly linked with FGC. Since FGC may well affect the nervous system beyond the reproductive tract (45, 46), it may be that as they age, cognition and mood are further affected.

Thus, there is a need to bring this understudied subgroup of postmenopausal African women with FGC into cognitive ageing research, specifically. By better understanding their cognitive ageing experiences along with their mood, we will be better able to assure their health as they age. The current review aimed to investigate what is already known about the cognitive and mood experiences of postmenopausal African women with FGC aged 50 years and above. Our primary research question was: *What is known about cognition and mood in postmenopausal African women with FGC?* We conducted a review of the literature to obtain the broadest perspective on what is currently known. Our work aims to highlight the gaps in the literature, opportunities for future research and the importance of evidence-based research in this population to strengthen health, community and systems that care for women with FGC.

## 2 Methods

### 2.1 Literature search strategy

We searched across three databases: PubMed, APA PsycInfo, and Google Scholar. PubMed was chosen for its extensive biomedical and life sciences literature and PsycInfo for its broad view of literature within the behavioural and social sciences (54, 55). Google Scholar was used to pick up any grey literature or qualitative studies missed by PubMed and PsycInfo (56, 57). Including grey literature in reviews has been shown to highly reduce publication bias, increase their comprehensiveness, and contribute to a more balanced overview of existing evidence on a topic (58). We included all results obtained from each search on PubMed and PsycInfo, even if they yielded beyond 50 articles. However, if one Boolean search on Google Scholar retrieved over 100 results, only the first 50 results were included for screening. Given that Google Scholar is known to return a large volume of information for most searches (57), including only the first 50 is standard practice for many reviews that have used the search engine to date. Overall, Google Scholar formed a powerful supplementary addition to our search strategy (59).

Boolean combinations of keywords related to FGC, cognition and its broad domains, mood, and ageing were used to identify relevant studies. Altogether, we used combinations of the following keywords: “female genital cutting”, “female genital circumcision”, “female genital mutilation”, “aging”, “ageing”, “Alzheimer's disease”, “mild cognitive impairment”, “attention”, “cognition”, “cognitive function”, “cognitive functioning”, “dementia”, “executive function”, “memory”, and “mood”. We used “mood” as a broad category representing affective states that encompass widespread forms of emotional and/or psychological conditions, such as depression or anxiety (60). We avoided the use of any keywords (e.g., “psychological disorders”) that may potentially bias our search to yield papers potentially pathologising the experiences of women with FGC. By including only one keyword for mood and multiple types for cognition and its main domains, we focused our review primarily on cognition and ageing, while also accounting for studies that have explored mood-related cognitive experiences of women with FGC [e.g., (61)] or any age-related mood experiences. Across all three databases, each Boolean combination was applied to search the full text to obtain a broad search, instead of searching by article title alone.

### 2.2 Study selection criteria & screening process

Inclusion criteria were: women with FGC, study cohorts including women aged 50 and older, local and/or immigrant African women, articles published in English, and all papers published before June 30, 2023. We did not limit our search based on whether their cognitive and mood experiences were explored qualitatively and/or quantitatively, article type/design, FGC type, or the age at which FGC was experienced. For any review articles obtained during our search, we further searched their included studies to screen more papers. Papers were excluded if they: did not include women with FGC aged 50 or over, investigated only/a majority of non-African FGC cohorts, published research studies without clear abstracts or summaries, were not written in English, or had broken Google Scholar links.

**Figure 1:**
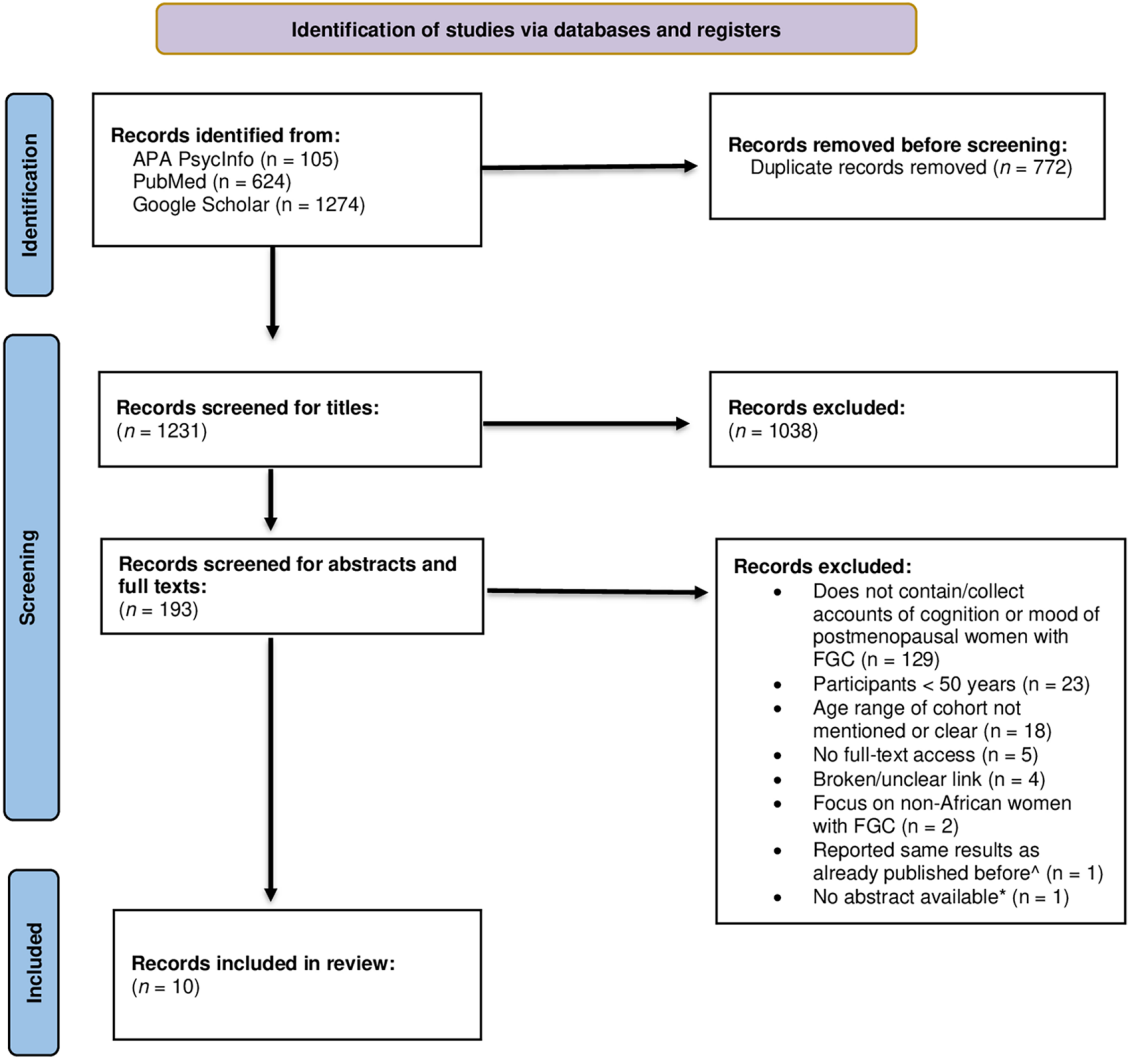
PRISMA flow diagram of the study selection process. APA, American Psychological Association; FGC, female genital cutting; ^, repeated the same results from the same cohort originally recruited in Vloeberghs et al. (64); *, only relevant for empirical research articles.

We used the Preferred Reporting Items for Systematic Reviews and Meta-Analysis (PRISMA) method (62) to document the search and screening processes as this method has been shown to improve the quality of literature reviews and aid in reducing bias [(63); Figure 1]. The abstracts of empirical research articles were first screened independently by two authors (R.K. & N.C.) before their full texts. For specific publication types and grey literature without abstracts (e.g., book chapters, commentaries, online blog posts, e-news articles), their full texts were directly assessed.

### 2.3 Data extraction

During the abstract screening process, the following information was extracted and put in table format using Microsoft Excel (65): article reference, study type and design, study focus, participant characteristics, age at FGC (in years), FGC type, relevant outcome measures, and relevant results. Upon compiling the information, all three authors resolved discrepancies in the extracted information and selected the final articles. All figures and tables were created using Microsoft Word (66).

## 3 Results

### 3.1 Overview of included records

Across the three databases, 2,003 records were identified. Upon removing duplicate records within and between databases, 1,231 records were then independently screened for titles by two authors (R.K. & N.C.). Any discrepancies in the independent title screenings were resolved by the same two authors, after which 1,038 articles were excluded, leaving 193 records. Next, the abstracts of those 193 records were screened using the eligibility criteria for the review. Once again, any discrepancies following these independent screenings were discussed and resolved, while bringing in the third author's (G.E.) input as necessary. After completing full-text screening in the same manner, 183 articles were deemed ineligible, leaving a final 10 papers included in our review (Figure 1). The summary characteristics of all included studies are compiled in Table 1 and relevant study-specific information is compiled in Table 2. Among the 10 records included, publication years ranged from 1980 to 2022, with 6 published in the last 10 years. The majority were qualitative studies with cross-sectional research designs; however, 3 were quantitative studies with either cross-sectional or case-control research designs. One article consisted of participant case reports.

**Table 1:** Overview of characteristics of included articles.

Article Characteristics (*n* = 10)	Proportions (*n*)
Published within the last 10 years (2013–2023)	6
Qualitative studies with cross-sectional design	4
Quantitative studies with cross-sectional design	3
Quantitative studies with case-control design	2
Case reports	1
Study cohort included women aged	
∼50–69	8
∼70–80	1
∼90	1
Majority FGC type within cohort	
Type III	6
Unreported	4
Cohort from	
The United States	2
The Netherlands	1
The United Kingdom	1
Norway	1
Germany	1
Egypt	1
Nigeria	1
Kenya	1
Ethiopia	1
Assessed	
Memory-related^a^ and mood-related experiences	5
Only memory-related^a^ experiences	4
Only mood experiences	1
Measures	
Interviewer-administered questionnaires about health symptom experiences	3
Semi-structured interview(s) about FGC event and experience	3
Both interviewer-administered questionnaires about health symptom experiences and semi-structured interviews about FGC experience	1
Structured interview schedule about FGC experience	1
Semi-structured interview schedule about health symptom experience	1
Self-reported questionnaires about health symptom experiences	1

^a^ Assessed the affective quality of cognition, but not cognition exclusively.

**Table 2:** Synopsis of findings of relevant articles.

Reference	Study type & design	Study focus	Participants	Age at FGC (years)	Type of FGC	Relevant outcome measures	Relevant results
Michlig et al. (67)	Quantitative, case-control	Psychological distress among Somali-American women via memories of FGC	879 immigrant Somali women with and without FGC living in the U.S. (15–90 years)^a^	*Range:* 0–15 *Mean:* 7.09	Type I = 25.37% Type II = 15.81% Type III = 27.65% Unknown FGC Type = 9.33% None = 18.32% Unknown = 3.53%	Questionnaires about adverse physical or psychological reactions during or immediately after FGC or any previously experienced traumatic event, and RHS-13; memory assessed by asking to recall	• Types I and III FGC associated with less distress symptoms. The same not found for Type II FGC • No association between the age of FGC and distress symptoms • 12% of the FGC cohort and 27% of the non-FGC cohort showed distress symptoms • 73.3% of the FGC cohort could not recall any adverse events related to FGC • If recalled, associated with distress • 63.6% of women who could recall any adverse event with FGC had experienced Type III FGC
Wulfes et al. (68)	Quantitative, cross-sectional	Physical and mental health of women with FGC in Germany seeking reconstructive surgery	112 immigrant women with FGC living in Germany (14–63 years)	*Range:* 0–21 *Mean:* 7.33	Type I = 18.75% Type II = 31.25% Type III = 50%	Questionnaires PHQ-2, GAD-2, PC-PTSD-5, SSGS shame and guilt subscales; memory assessed by asking to recall	• 36.6% suspected depression • 48.2% suspected anxiety • 55.4% suspected PTSD • Feelings of guilt and age of FGC significant predictors for PTSD symptoms • Current age and FGC type not significant predictors for PTSD symptoms
Assaad (69)	Case reports	Egyptian women's views on how FGC affects them, their attitudes toward it, and how their attitudes may be changing	4 local Egyptian women with FGC (22–60 years)	*Range:* 8–9 *Mean:* 8.25	Type I = 25% Type II = 25% Unknown = 50%	Structured interview of 57 questions, including about FGC; memory assessed by asking to recall	• One 60-year-old participant with Type II FGC at 8 years had vivid autobiographical memory of her FGC event • Could provide detailed accounts of her experiences a week before, the day of, and a week after FGC • Recalled feeling deceived by her mother and by woman performing FGC • Reported intense fear during and immediately after event
Gacheru (70)	Qualitative, cross-sectional	Kenyan Kikuyu women's experiences and perceptions of FGC in the context of its emotional impact	12 local Kikuyu (Kenyan) women with FGC (35–55 years)	*Range:* 10–15	NR	Semi-structured interviews with open-ended questions asking to: describe their experience with FGC, investigate whether they can recollect their FGC event, see what they remembered, and what their current feelings are; memory assessed by asking to recall	• Vivid memories of FGC event • Recall of its effect before, during, and immediately following the event • Subsequent feelings of loss of trust, unworthiness, and incompleteness
Omigbodun et al. (71)	Qualitative, cross-sectional	Nigerian women with FGC's psychological experiences	38 local Izzi (Nigerian) women with FGC (18–60 years)	NR	NR	Interviews (1–2 h) questions from the adapted MINI; memory assessed by asking to recall	• Majority remembered their FGC experience • Feelings of anger, sadness, shame, and embarrassment before their FGC. • Happy anticipation closer to the day of their FGC event. • During FGC, feelings of intense fear. • Immediately following event, some expressed eventual happiness, while others expressed intense emotional turmoil reminiscent of PTSD symptoms.
Lockhat (72)	Quantitative, cross-sectional	Prevalence rate of psychological distress, physical challenges, complications, and other variables in immigrant Sudanese and Somali women with FGC	55 immigrant Sudanese and Somali women with FGC living in the U.K. (21–60 years)	*Range:* 2–13 *Median:* 7	Type I = 18.18% Type II = 20% Type III = 61.82%	Questionnaire about stressful life events HADS, IES, CAPS-1, semi-structured questionnaire asking questions about the FGC event; memory assessed by asking to recall	• 96.36% participants remembered their FGC event • Vivid accounts of their experience before, during, and following the event: almost half reported as negative, others reported as positive • 3.63% reported as neutral • Type III FGC; more likely to have anxiety and depression • 7.2% met criteria for current PTSD • 27.3% met criteria for lifetime PTSD • More than half did not report their FGC as a stressful life event.
Schultz & Lien (73)	Qualitative, cross-sectional	Type of quality of psychological care provided to African girls before, during and after FGC. Description of the beliefs systems underlying FGC-related care provided in Gambia	20 immigrant African (Gambia, Somalia, and other unnamed neighbouring countries) women with FGC living in Norway (32–60 years)	NR	Most had Type III, others unknown	Semi-structured interviews about childhood experiences and FGC narratives; memory assessed by asking to recall	• Clear recollections of their FGC event. • Painful memories of FGC. • Recalled forming short-lived negative relationships with their mothers if they played an active role in FGC event. • Negative emotions at the time: such as fear, numbness, disbelief, betrayal, sadness, loss of trust, and anger • Some recount happiness and pride.
Dahlen (74)	Qualitative, cross-sectional	Life experiences of first-generation Ethiopian immigrant mothers in the US in the context of FGC	9 immigrant Ethiopian women with and without FGC living in the U.S. (late 20s-mid 50s)	NR	Unknown FGC Type = 77.78% None = 22.22%	Three in-depth semi-structured interviews exploring their current lived experiences and narratives about FGC; memory assessed by asking to recall	• 44.44% could not recall their FGC event. • 33.33% could recall the event in detail. • 11.11% recalled it vividly. • One (in her mid-50s) with vivid memory recalled feeling upset, helpless, screaming, and crying during FGC event. • Another (in her mid-50s) had limited memory but stated not feeling proud of her parents for having her experience FGC.
Köbach et al. (75)	Quantitative, case-control	Psychopathological sequelae of FGC in Jijiga, in the context of stress-related variables	165 local African women with and without FGC living in Jijiga, Ethiopia (13–80 years)	*Type I FGC* *Mean:* 3.1 *Type II/III FGC Mean:* 7.6	Type I = 36.36% Type II = 4.85% Type III = 47.88% None = 10.91%	Questionnaires administered: 45-item checklist about FGC, CFV, PDS, PSS-I, HSCL-25, and M.I.N.I.; memory assessed by asking to recall	• 18% of women with Type II/III FGC and 6% of women without FGC met PTSD criteria. • 12% of women with Type II/III FGC met major depressive disorder criteria. • Significant association between Type II/III FGC and PTSD, depression, and anxiety symptoms. • 92% of participants who remembered their FGC event reported experiencing intense fear and/or helplessness during the event.
Vloeberghs et al. (64)	Quantitative, cross-sectional	Psychosocial and relational challenges of African immigrant women with FGC living in the Netherlands	66 immigrant women from Somalia, Sudan, Eritrea, Ethiopia, and Sierra Leone with FGC living in the Netherlands (18–69 years)	*Range:* 8 months–16 years *Mean:* 6.4	Type I = 31.82% Type II = 13.64% Type III = 53.03% Unknown FGC Type = 1.52%	Questionnaires administered: HTQ-30 and HSCL-25, semi-structured interviews including questions about FGC event; memory assessed by asking to recall	• 50% had vivid memory of their FGC event. • 19.69% had some memory. • 21.21% had no memory. • Vivid memories and those with Type III FGC more likely to report higher PTSD symptoms, and anxiety and depression symptoms. • Those older at the time of FGC had significantly more PTSD symptoms, but not more anxiety or depression symptoms. • 17.5% had an indication of PTSD. • 31.7% met the cut-off for anxiety. • 34.9% met the cut-off for depression. • Independent of African community, recurrent bad memories, and nightmares in relation to FGC event reported. • Independent of African community, feelings of fear, powerlessness, anger, shame, and guilt in relation to their FGC event reported. • Pride post-FGC rarely reported.

CAPS-1, clinical rating scale for assessing current and lifetime post-traumatic stress disorder; CFV, checklist of childhood familial violence; FGC, female genital cutting; GAD-2, generalized anxiety disorder scale-2; HADS, hospital anxiety and depression scale; HSCL-25, Hopkins symptom checklist-25; HTQ-30, Harvard trauma questionnaire-30; IES, impact of event scale; M.I.N.I., mini-international neuropsychiatric interview; MINI, McGill Illness narrative interview; NR, not reported; PC-PTSD-5, primary care post-traumatic stress disorder screen for diagnostic and statistical manual of mental disorders - 5th edition; PDS, post-traumatic stress diagnostic scale; PHQ-2, patient health questionnaire-2; PSS-I, PTSD symptom scale-interview; PTSD, post-traumatic stress disorder; RHS-13, refugee health screener-13; SLE, stressful life events; SSGS, state shame and guilt scale.

^a^ 69.5% were born in Somalia, 19.0% in Kenya, 6.3% in other African or Middle Eastern countries, and 5.2% in the U.S. or Europe.

Across all the studies, the number of African women investigated (with FGC, without FGC, or both) ranged from 9 to 879, with ages ranging from 13 to 90 years. Five studies clearly indicated the number of postmenopausal women with FGC; in these, they ranged from 4.5% to 25% of the total study cohort. In the other five studies, it was impossible to discern who was and was not postmenopausal. Although they constituted a minority of their cohorts, 6 studies included women aged approximately 50–69 years. Five studies reported the mean age at FGC as between 3 and 8.25 years. One reported their median age at FGC to be 7 years, one reported the age range at FGC to be 10–15 years, and three did not report age at FGC. Among studies that collected participants' age at FGC, only one reported the ages at FGC for each included participant.

In six studies, a majority of the FGC cohort had Type III FGC. Women across these studies hailed from a total of 16 African countries: Somalia, Sierra Leone, Sudan, Eritrea, Ethiopia, Egypt, Kenya, Nigeria, Gambia, Guinea, Burkina Faso, Senegal, Ivory Coast, Liberia, Chad, and Djibouti. Six studies investigated the experiences of immigrant African women with FGC living in Western countries, while four investigated their experiences within their local African contexts.

Five studies investigated mood and memory experiences related to their FGC experiences. Among those investigating memory, all focused on the affective quality of FGC memories and did not focus on other aspects of cognition (e.g., memory *per se*, attention, or executive function). Nine studies conducted interviewer-administered questionnaires assessing their physical or psychological health symptoms or semi-structured interviews about their FGC event, experiences, and feelings. Altogether, seven studies aimed to investigate how FGC affected the lives of African women and collected details about their FGC event; two collected information on the FGC event and the immediate emotional impact it had on them, and one collected information on the FGC event and explored their views/attitudes towards the practice.

Across most studies, it was unclear whether the cognition- and/or mood-related experiences and findings also applied to women with FGC aged 50 and above. The results from the included studies are listed according to study type and design below.

### 3.2 Qualitative studies with cross-sectional research designs

A total of four studies analysed the experiences of African women with FGC using qualitative methods. One study (73) analysed the experiences of 20 African immigrant women with FGC living in Norway (aged 32–60 years) using semi-structured interviews to capture details about their FGC experience. Although no descriptive statistics was reported, the authors indicated that most women in their cohort had Type III FGC. The interviews revealed that all participants vividly recalled the series of events that took place on the day of their FGC. One participant stated “…it is all recorded down to every detail as if it was a film. My problem is not remembering but trying to forget” (p. 212). Few also remembered their mothers playing an active role in their FGC experience and recalled their relationships with their mothers being negatively affected in the initial weeks post-FGC. Specifically, they described experiencing negative reactions such as anxiety, fear, numbness, disbelief, betrayal, and anger towards their mother. Many also reported feeling frustration, sadness, and loss of trust. However, they reported these negative reactions as being short-lived. In contrast, some participants reported positive reactions to certain aspects of FGC, such as happiness when receiving gifts and praises, and pride once they completed the ritual.

Similarly, four of nine first-generation Ethiopian immigrant mothers reported being unable to recall their FGC experience (74). This inability to recall the event was attributed to their FGC being performed during infancy, though their exact ages at FGC were not reported. Three participants could recall their experience, though in sparse detail, estimating that their FGC occurred between the ages of three to nine. One participant in their mid-50s (pseudonym “Mulu”) was able to recall her experience with only some detail and reported experiencing it as a young child. However, whatever she could recall held negative connotations. She mentioned that she did not feel heard as a child when she tried to voice her opinion on the ritual. She stated, “If they had asked me, I wouldn't have let them do it” (p. 71) and later said, “I'm not proud of my parents doing that to me” (p. 131). One participant in her mid-50s (pseudonym “Telile”), who also experienced FGC as a young child, did recall her FGC experience quite vividly and said, “You remember in your ears what happened” (p. 69). She also remembered feeling upset, screaming, and crying during her FGC experience, and said, “I know it was going to happen…that's why I refuse and I fight” (p. 131). She also recalled feeling helpless.

A qualitative study (70) examining the psychological and emotional experiences of 12 local Kenyan women (aged 35–55 years) found that their memories of FGC were fresh. A vividness was observed across participants, despite experiencing FGC at different ages ranging from 10 to 15 years. Some expressed extreme fear before their ritual; though only one participant (P5) in their study, who was between 55 and 60 years old, recalled feeling excited beforehand. Many participants also described experiencing intense and long-term loss of trust, and feelings of unworthiness and incompleteness later in life.

A qualitative study (71) used an adapted version of the McGill Illness Narrative Interview Schedule [MINI; (76)] to understand the embodied psychological experiences of 38 local Nigerian women with FGC (aged 18–60 years). Virtually all participants experienced intense negative psychological feelings such as anger, sadness, shame, and embarrassment before their FGC, as they recalled being mocked and humiliated for not being cut during this period. On the other hand, some recalled experiencing positive emotions, particularly happy anticipation closer to the day of their FGC. During the ritual, many described feeling intense fear. After FGC, participants described emotions ranging from positive (e.g., happiness from escaping stigma and gaining a respectful status), negative (e.g., betrayal, anger, and fear of dying), and mixed emotions. In terms of their long-term experiences post-FGC, some experienced happiness due to not facing any complications following FGC; whereas others experienced intense emotional turmoil linked to their complications.

### 3.3 Qualitative case reports

One article (69) described case reports of four local Egyptian women with FGC (aged 22–60 years) via individual structured interviews. The case report of one 60-year-old Egyptian woman (pseudonym “Camilia”) who had FGC at age 8, revealed that she remembered her FGC experience “as if it happened yesterday” (p. 11) and was able to provide detailed accounts of her experience, starting from the week before her cutting, the day of her ritual, and approximately a week post-cutting. She also remembered what others around her (specifically, her mother and the woman who performed the practice) remarked about the practice and vividly recalled feeling deceived by them. She also detailed other negative emotions, particularly fear, that she experienced during and immediately after FGC.

### 3.4 Quantitative and mixed-method studies with cross-sectional research designs

Three studies used quantitative methods to investigate FGC and related experiences among a cross-sectional cohort. One mixed-methods (64) study assessed the long-term psychological and relational consequences of FGC among 66 immigrant African women with FGC living in the Netherlands (aged 18–69 years) using semi-structured interviews and two interviewer-administered questionnaires about their PTSD, depression, and anxiety symptoms, namely: the Harvard Trauma Questionnaire-30 [HTQ-30; (77)] and the Hopkins Symptom Checklist-25 [HSCL-25; (78)]. The findings revealed differences in FGC recollection; 33 participants had a vivid memory, 13 had some memory, and 14 had no memory. Those who recalled their FGC event and those who experienced Type III FGC reported significantly more PTSD, anxiety, and depression symptoms. Those older at the time of FGC reported significantly more PTSD symptoms, but not more anxiety or depression symptoms. All women with FGC hailing from different African communities in the study cohort reported experiencing recurrent bad memories and nightmares of their FGC events, as well as fear, feelings of powerlessness, tension, apathy, exclusion, anger, shame, and guilt. Only one participant reported experiencing pride after FGC. Eleven participants showed an indication of PTSD, 20 met the cutoff for anxiety, and 22 met the cutoff for depression. There were no significant differences in these outcomes based on age.

Another study (72) looked at the experiences and feelings of 55 immigrant Sudanese and Somali women with FGC living in the U.K. (aged 21–60 years). The authors used the following set of mood-related questionnaires: the Hospital Anxiety and Depression Scale to measure stressful life events [HADS; (79)], the Impact of Event Scale [IES; (80)] to measure the intrusions and avoidance symptoms of PTSD, the Clinical Rating Scale for Assessing Current and Lifetime PTSD-1 [CAPS-1; (81)], and a semi-structured questionnaire asking about their FGC event. Two participants in the study were unable to recall any aspect of their FGC event: one experienced Type I FGC (Sunna), and the other experienced Type III FGC (Pharaonic). Among the 53 who remembered their FGC experience, most were able to provide vivid accounts of events that took place and their feelings before, during, and directly after the ceremony. Their feelings before FGC widely varied, including excitement and/or fear. Immediately after FGC, there was ample variation in their overall memories of thoughts and feelings about the FGC experience, ranging from contentment and pride to indifference, anger, sadness, and confusion. The authors' qualitative analyses of those who remembered their FGC event revealed that 26 participants reported their overall experience to be negative, 25 as positive, and two as neither negative nor positive. In terms of their mood-related experiences, they found that women with Type III FGC were more likely to have higher anxiety and depression scores relative to others with Type I. Twenty-nine participants did not report FGC as a stressful life event and 26 did. Only four participants met the criteria for current PTSD and 15 met the criteria for lifetime PTSD.

Finally, another study (68) examined the physical and mental health characteristics associated with PTSD symptoms among 112 immigrant women with FGC living in Germany (aged 14–63 years). Authors administered the following relevant self-report screening instruments via the Patient Health Questionnaire-2 [PHQ-2; (82)] to measure depression symptoms, the Generalized Anxiety Disorder Scale-2 [GAD-2; (83)] to measure anxiety symptoms, and the Primary Care PTSD Screen for the Diagnostic and Statistical Manual of Mental Disorders-5th Edition [PC-PTSD-5; (84)] to screen for PTSD. One hundred and seven participants hailed from Africa, while the remaining were from Qatar, Great Britain, and Iraq. Scores obtained above the cutoff on these screening tools were said to show 'suspected' symptomatology for those disorders. They also assessed trauma-associated shame and guilt by using some subscales within the State Shame and Guilt Scale [SSGS; (85)]. Their findings showed that 41 participants had suspected depression, 54 had suspected anxiety disorder, and 62 had suspected PTSD. The age at FGC was a significant predictor for PTSD symptoms, such that older age at FGC was associated with higher PTSD symptomatology. Feelings of guilt were found to be a significant predictor of PTSD symptoms, while feelings of shame were not. No significant differences in PTSD symptomatology were found based on current age and FGC type.

### 3.5 Quantitative studies with case-control research designs

Two quantitative studies used case-control research designs to investigate the experiences of African women with FGC relative to those without FGC. One of these studies (67) analysed whether FGC was associated with psychological distress among 879 Somali immigrant women with FGC living in the United States. The control group consisted of Somali American women without FGC. All participants ranged from 15 to 90 years. The FGC group was also asked if they could recall any adverse physical or psychological reactions at the time of or immediately following their FGC event. Their distress symptoms were recorded using the Refugee Health Screener-13 [RHS-13; (86)]. Participants' ages were controlled across all statistical analyses conducted. Findings showed that most women with FGC did not recall any adverse events experienced either during or immediately after their FGC; though they found that a majority of women who were able to recall any adverse events had experienced Type III FGC. However, those with Types I and III FGC reported significantly fewer distress symptoms than Somali American women without FGC. There was no significant association between experiencing Type II FGC and distress symptoms. Only 12% of participants from the FGC cohort showed clinically significant distress symptoms, whereas the non-FGC cohort had slightly more participants showing distress symptoms (27%). No association between the age at FGC and distress symptoms was found. Moreover, participant's ability to recall any adverse events during FGC was strongly associated with experiencing clinically significant symptoms of distress.

Using a case-control design and a mixed methods approach (75), PTSD and other stress-related variables were investigated among 165 local African women with and without FGC living in Ethiopia (13–80 years). The following were verbally administered: traumatic experiences using the Checklist of Childhood Familial Violence [CFV; (87)] and the event checklist within the Post-Traumatic Stress Diagnostic Scale [PDS; (88)], PTSD symptoms using the PTSD Symptom Scale-Interview [PSS-I; (89)], depression and anxiety symptoms using the HSCL-25 (78), and the diagnostic status of major depression, suicidal ideation, drug abuse, and psychotic disorders using the Mini-International Neuropsychiatric Interview [M.I.N.I.; (90)]. In addition, the FGC cohort's experiences with the ritual were also explored via a 45-item checklist constructed by the authors. Women with FGC who could not remember specific details about their FGC experience were excluded from analyses. This was found to be the case for women who experienced FGC at 3 years old, for one participant with Type I FGC, and two participants with Type II/III FGC. Ninety six women with FGC who remembered their FGC experience, regardless of FGC type, reported experiencing intense fear and/or helplessness during the ritual. Sixteen women with Type II/III FGC and one without FGC met the criteria for PTSD. Eleven women with Type II/III FGC met the criteria for major depressive disorder. All women without FGC did not meet the criteria for depression, and all except one did not meet criteria for PTSD. Women with Type I FGC neither met the criteria for PTSD nor depression. The authors also found a significant association between having Type II/III FGC and a greater vulnerability to PTSD symptoms, depression, and anxiety symptoms.

## 4 Discussion

The current review aimed to identify and summarise what is already known about the cognition and mood experiences of local and immigrant postmenopausal African women with FGC. Upon completion of the screening process, 10 articles were included in the current review. No studies to date have exclusively investigated the experiences of a postmenopausal African FGC cohort aged 50 years and above. Likewise, no study has explored cognition, ageing, or memory in any context beyond their FGC experience. Participants were specifically asked to recount or answer questions about their FGC ceremony and often, those were paired with questionnaires about pathological conditions like anxiety, depression, and PTSD. In terms of cognition, qualitative accounts revealed that African women with FGC tend to hold vivid episodic autobiographical memories of the ceremony, including the series of events that took place, their feelings about other people involved, and their feelings leading up to, during, and after FGC. Among those who remembered the ceremony, feelings of anger, guilt, shame, fear, and helplessness were common, while feelings of indifference, happiness, or pride were less reported. Despite these negative feelings and high levels of psychological distress- and disorder-related symptoms reported, only a minority had PTSD, anxiety, depression, or psychological distress when interviewer-administered measures were used. In general, those who had more severe FGC types or were older at the age of FGC were more likely to have higher PTSD and psychological distress symptoms.

We aimed to capture different aspects of cognition with our review (i.e., memory, executive function, and attention). However, our searches only yielded articles that focused on autobiographical memory via qualitative retrospective accounts and did not analyse them in the context of memory research. No quantitative cognitive, neuropsychological, and/or neuroimaging measures were used to complement these results and further analyse participants' memories. Indeed, no study has analyzed other aspects of memory such as verbal episodic memory or associative memory which are impacted by dementia. Qualitative retrospective accounts, although valuable in capturing the nuances of individual lived experiences, can be prone to recall bias or misclassification bias if used alone (91). Currently, the only study to date using a neuropsychological assessment tool is a study investigating cognition in young African women with FGC aged 15–40 years (52). In this study, they found that a diagnosis of PTSD among African women with FGC was significantly associated with the presence of significant memory problems, relative to those without FGC.

The vast majority of studies found in our larger search focused on FGC's reproductive outcomes. Once these were excluded, the included studies investigating the memory experiences of older women with FGC still focused on the affective quality of their FGC memories and adverse psychological outcomes. Although they included older cohorts within their study cohorts, we found that no studies were conducted with postmenopausal women exclusively. Of the 10 studies included, older women formed a minority of the study cohort. There were none or unclear categorisations of these women's ages in most of the studies. Due to this, it was also unclear whether the cognition- and/or mood-related findings found also generalised to older women with FGC and were similar to those in the cohort that were of reproductive age. Therefore, future studies should prioritise investigating the cognitive experiences of postmenopausal African women with FGC to holistically understand how the practice might affect their cognition as they age.

This lack of cognitive ageing studies of local and immigrant postmenopausal African women with FGC is a serious gap in the literature, especially since existing research shows that some, at least, have the markers of early cardiovascular disease, depression, and possibly, chronic pain – all associated with late-life cognitive decline (45–47, 92, 93). Longitudinal studies to date have shown that sub-Saharan women tend to have substantially worse cognitive health and a significantly steeper age gradient in cognitive ability, relative to sub-Saharan men, over time (94). Similarly, studies have found that African American women tend to experience steeper rates of cognitive decline relative to White and Hispanic women and men (95). Thus, there is a dire need to study cognitive ageing in postmenopausal women with FGC to fully determine the prevalence of neurodegenerative diseases, healthcare needs, and quality of life.

Our search did not find articles on cognition. Rather, the focus was on memory of the FGC ceremony and events before and after which were vivid, suggesting that their autobiographical memory is intact. This was especially the case for women older than age three when they had FGC or had Type III FGC. This is consistent with existing literature showing that by the age of three children can start to retain autobiographical episodic memories (96, 97). This trend is also consistent with general ageing research demonstrating that both male and female older adults tend to subjectively report high levels of vividness for remote autobiographical memories, sometimes even higher, relative to young adults (98). Reviews have shown that while there is an age-related decline in the level of detail provided by older adults, their overall gist representation of the event remains (99, 100).

Among the included studies that reported their cohort's FGC type, most women, regardless of FGC type, tended to remember strong feelings of either fear, anger, excitement, or happiness. Thus, their FGC event was likely an emotionally charged memory for them and likely, an emotionally charged event. Mixed-sex studies on memory in older adults reveal that although they tend to produce greater semantic details and fewer episodic details for emotionally charged autobiographical events relative to younger adults, the overall number of details recalled for such events remains strong with age (101).

While episodic autobiographical memories were related, much of the focus of the 10 studies was on women's FGC subjective memories, particularly their affective experiences. Specifically, they reported high levels of negative emotions such as anger, shame, fear, helplessness, and guilt, in relation to their FGC. Although in one study, as many as half of the participants reported positive or neutral emotions related to their FGC event. To date, mixed-sex ageing research has shown evidence for individuals' tendency to pay attention to positive information and avoid negative information as they age (102, 103). Interpreted in the context of this “positivity effect”, women in the reported studies were specifically asked about FGC and not about general recollection of life events, so it is unclear whether the “positivity effect” pertains to them. Mixed-sex studies have shown that older adults with MCI and dementia tend to experience more negative emotions (104, 105); however, again, there is no way of knowing the cognitive state of the participants in the studies cited here as it was not exclusively explored. Using relevant quantitative measures, future studies should investigate whether there is actually a positivity or negativity bias in FGC-related recall. This is especially important given that experiencing intense negative emotions increases the risk of developing depression, which in turn is a risk factor for dementia (106, 107). There may be more cause for concern, given that older women, in general, are more likely to be at risk of developing depression than older men (106, 108, 109). Furthermore, future studies should also aim to analyse the valence of this cohort's qualitative accounts using well-validated quantitative measures and, accordingly, report what proportions of their cohorts recalled experiencing positive, negative, and/or neutral emotions to various aspects of their FGC as the negative bias may be built into the study, itself, much like studies of menstruation (110).

In addition to memories of FGC, studies included measures of psychological distress. These revealed little to no findings of psychological distress, PTSD, depression, and anxiety among women with FGC, regardless of study type and design. When control groups were present, the outcomes of women with FGC did not significantly differ from women without FGC. Given that research has shown increased levels of depression, anxiety, and clinical distress to be strongly associated with lower levels of cognitive health as individuals age (111, 112), with the decline in cognitive health often being substantially worse for women (94), future research should continue to explore age-related changes in the mood experiences of postmenopausal women with FGC and correlate those with cognitive outcomes. It is, however, critical to do this research without pathologising women's mood experiences and instead, attempting to uphold consistency with each African population's local terms, concepts, and views of mental health (113).

In sum, we have found a dearth of knowledge about ageing women with FGC whether locally or in countries to which they have immigrated. For their health, it is important to bring this radically understudied group of postmenopausal African women with FGC into ageing and dementia research. Doing this will lead to a better understanding of their evolving age-related cognition, potential etiologies of dementia, and cognitive health needs. Receiving evidence-based, sensitive ageing care may also serve to reduce the known barriers they have with accessing and/or receiving appropriate healthcare in Western contexts. Further research is needed to analyze the crucial implications on policy and practice related to aging with FGC.

## 5 Limitations

The main limitation of this review is that the dearth of literature on ageing women with FGC in any context, as well as the primary focus on FGC and reproductive health, drastically limited the number of papers recovered. This is an important finding since it opens the necessity of including these women in studies of health beyond the reproductive system, including cognitive ageing. Given that there were no studies on our topic, we did not conduct a systematic review and/or a meta-analysis. Instead, we reached more broadly for any study that included older women with FGC and asked them about their memories; a narrative review facilitated a wider search on this topic. Another limitation is that we used Google Scholar as one of our search databases. Research has shown Google Scholar to be inappropriate as a principal search engine for reviews as it lacks reproducibility and transparency in its search algorithm as well as around the size of its database (59, 114). Nonetheless, studies evaluating the search engine have shown that although it is considered unsuitable for primary review searches, it is a suitable and often helpful supplementary source of evidence, especially for grey literature (57). Thus, Google Scholar allowed for the widest search possible. Indeed, seven out of the ten papers, some of which were published theses/dissertations, included in our review were obtained from our Google Scholar searches. Using it and not finding any other reports underscores the dearth of any kind of study on cognition in ageing women with FGC. A strength of this review is that it is the first to search the literature for cognitive ageing studies in older women with FGC, marking the first step to organising existing literature on this topic. We also used the PRISMA methodology in our search to improve rigour and avoid bias (62).

## 6 Conclusion

The current review revealed some evidence for the cognitive and mood experiences of local and immigrant postmenopausal African women with FGC, primarily around their FGC experience. To date, no studies have exclusively investigated cognitive ageing in this group of women. Instead, ageing women with FGC have been integrated into cohorts consisting of a wide range of ages with the older women constituting a minority. The questions asked of them only reveal their accounts of autobiographical memory, specifically with respect to FGC. These studies reveal that most, but not all, older women with FGC report vivid memories of FGC. However, since the studies do not focus on capturing memory *per se*, studies do not report details about their memory abilities, leaving open how much of their cognition is actually affected. When these studies include questions about mood, they reveal low incidences of PTSD, depression, and anxiety symptoms in the face of reported feelings of anger, guilt, and shame. With increasing life expectancy, AD is becoming a concern in Africa as well as in the countries to which Africans have immigrated (8). This demands that international health care policy begin to consider the cognitive health of Africans; and especially, female Africans with early life adversity, so that we can determine the earliest, most efficacious times for treatment. In this sense, future research should explore their cognitive health in-depth using culturally sensitive and mixed-method approaches.

## Data Availability

The original contributions presented in the study are included in the article/Supplementary Material, further inquiries can be directed to the corresponding author.
